# Are Mast Cells MASTers in Cancer?

**DOI:** 10.3389/fimmu.2017.00424

**Published:** 2017-04-12

**Authors:** Gilda Varricchi, Maria Rosaria Galdiero, Stefania Loffredo, Giancarlo Marone, Raffaella Iannone, Gianni Marone, Francescopaolo Granata

**Affiliations:** ^1^Department of Translational Medical Sciences (DiSMeT), Center for Basic and Clinical Immunology Research (CISI), University of Naples Federico II, Naples, Italy; ^2^Department of Public Health, University of Naples Federico II, Monaldi Hospital Pharmacy, Naples, Italy; ^3^Institute of Experimental Endocrinology and Oncology “Gaetano Salvatore” (IEOS), National Research Council (CNR), Naples, Italy

**Keywords:** angiogenesis, cancer, inflammation, lymphangiogenesis, mast cells

## Abstract

Prolonged low-grade inflammation or smoldering inflammation is a hallmark of cancer. Mast cells form a heterogeneous population of immune cells with differences in their ultra-structure, morphology, mediator content, and surface receptors. Mast cells are widely distributed throughout all tissues and are stromal components of the inflammatory microenvironment that modulates tumor initiation and development. Although canonically associated with allergic disorders, mast cells are a major source of pro-tumorigenic (e.g., angiogenic and lymphangiogenic factors) and antitumorigenic molecules (e.g., TNF-α and IL-9), depending on the milieu. In certain neoplasias (e.g., gastric, thyroid and Hodgkin’s lymphoma) mast cells play a pro-tumorigenic role, in others (e.g., breast cancer) a protective role, whereas in yet others they are apparently innocent bystanders. These seemingly conflicting results suggest that the role of mast cells and their mediators could be cancer specific. The microlocalization (e.g., peritumoral vs intratumoral) of mast cells is another important aspect in the initiation/progression of solid and hematologic tumors. Increasing evidence in certain experimental models indicates that targeting mast cells and/or their mediators represent a potential therapeutic target in cancer. Thus, mast cells deserve focused consideration also as therapeutic targets in different types of tumors. There are many unanswered questions that should be addressed before we understand whether mast cells are an ally, adversary, or innocent bystanders in human cancers.

## Introduction

Mast cells were first identified in human tumors and named by Paul Ehrlich (1, 2). These cells are present in all classes of vertebrates, and it has been estimated that they have emerged >500 million years ago, long before the development of adaptive immunity (3). Mast cells are distributed throughout nearly all human tissues and often in close proximity to epithelia, fibroblasts, blood and lymphatic vessels, and nerves (4).

Human mast cells form a heterogeneous population of cells with differences in their ultrastructure, morphology, mediator content, and surface receptors (4, 5). Human mast cells derive from CD34^+^, CD117^+^ pluripotent hematopoietic stem cells, which arise in the bone marrow (6). Mast cell progenitors enter the circulation and subsequently complete their maturation in tissues. These cells store and release upon activation a wide spectrum of biologically active mediators that individually have been shown to have potential positive or negative effects on various target cells (7). Increasing evidence indicates that mast cells act as sentinels of the surrounding environment, with the capacity to rapidly perceive tissue insults and initiate biochemical programs of inflammation or repair.

**Figure 1:**
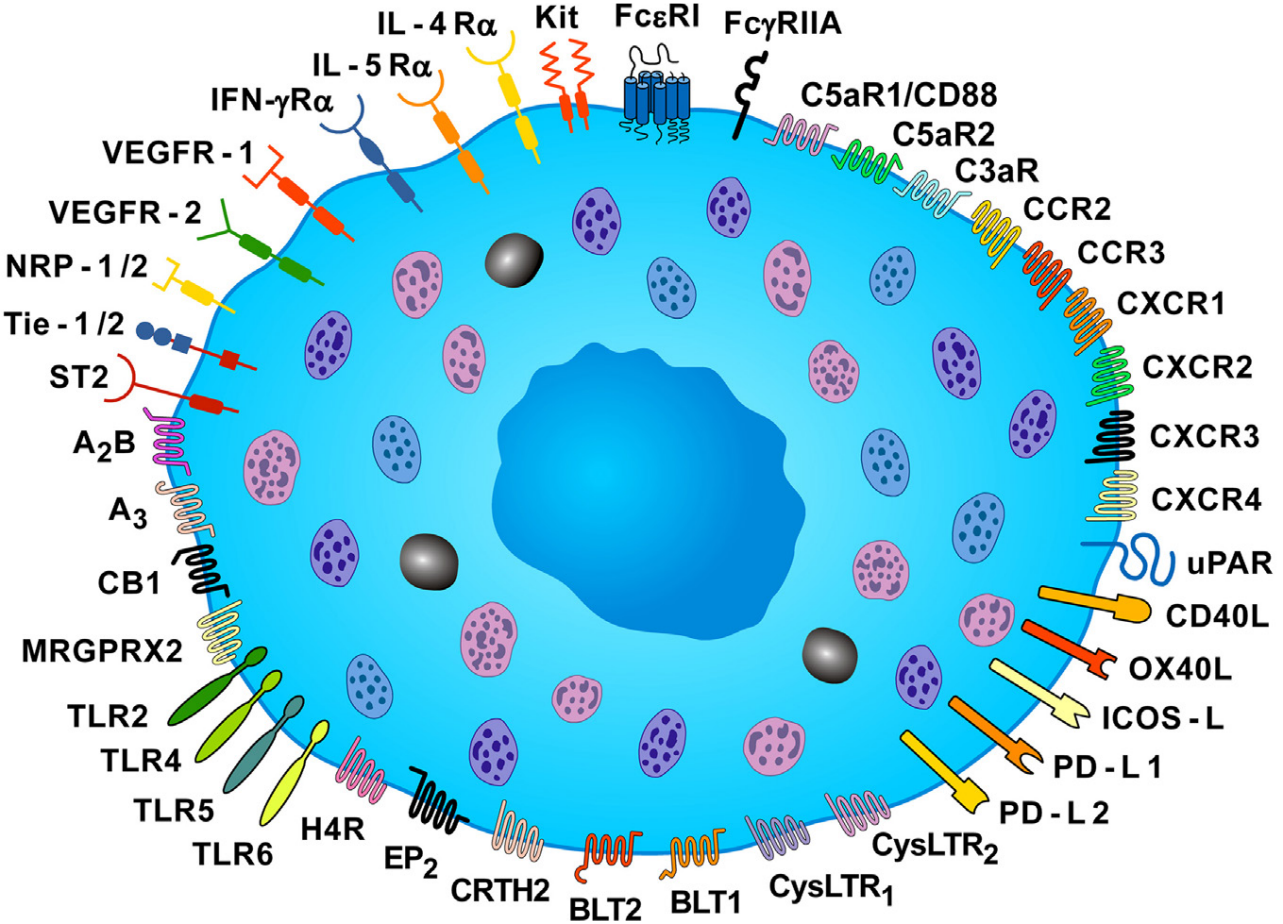
**Some of the surface receptors expressed by human mast cells**. Human mast cells express the tetrameric high-affinity receptor for IgE (FcεRI) and the FcγRIIA, and their cross-linking induces the release of pro-inflammatory and immunomodulatory mediators. Mast cell expresses the KIT receptor (CD117), which is activated by stem cell factor. These cells express a plethora of receptors, such as toll-like receptor (TLR) 2, TLR4, TLR5, TLR6, receptors for chemokines (CCR2, CCR3, CXCR1, CXCR2, CXCR3, and CXCR4), two receptors for cysteinyl leukotriene (CysLTR1 and CysLTR2), two leukotriene B_4_ receptors (BLT1 and BLT2), the prostaglandin D_2_ receptor (CRTH2), the prostaglandin E_2_ receptor (EP_2_), the cannabinoid CB1 receptor, two adenosine receptors (A_2B_ and A_3_), and histamine H4 receptor (H4R). Mast cells express receptor for various cytokines (IL-4Rα, IL-5Rα, IFN-γRα, ST2). The MAS-related G protein coupled receptor (MRGPRX2) can be activated by neuromuscular blocking drugs, neuropeptides (SP and VIP), and eosinophil cationic proteins (MBP and EPX). These cells also express receptors for vascular endothelial growth factors (VEGFR1 and VEGFR2), and VEGFR co-receptors, neuropilin-1 and neuropilin-2 (NRP1 and NRP2), for anaphylatoxins (C5aR1/CD88, C5aR2, and C3aR), and the high-affinity urokinase plasminogen activator receptor (uPAR). Human mast cells also express co-receptors for T-cell activation [CD40 ligand (CD40L), tumor necrosis factor superfamily member 4 (OX40L), inducible costimulator ligand (ICOS-L), programmed death ligands (PD-L1 and PD-L2)] [Slightly modified with permission of Springer Nature from Borriello et al. (15)].

Mast cells are activated not only by IgE (8), specific antigens (5), and superallergens (9, 10), the main mechanisms which account for their function in allergic disorders, but also by a plethora of immunologic and non-immunologic stimuli (11–14). Figure 1 schematically illustrates the constellation of surface receptors expressed by human mast cells.

Mast cells and their mediators have been canonically associated with a detrimental role in allergic diseases (4, 5), but these cells can induce a protective immune response of the host against noxious substances (16, 17), viral (18) and microbial pathogens (19). Interestingly, epidemiological (20, 21) and experimental studies (22) indicate an inverse association between IgE-mediated allergies and cancer, implying tumor-protective effect of IgE.

The initiation and progression of cancer are multistep processes characterized by the accumulation of a variable number of genetic and epigenetic alterations (23). The immunosurveillance system recognizes and eliminates mutant cells constantly generated (24). However, immune-resistant cancer cells can slip through this system and proceed to develop tumors (25).

Normal microenvironment [immune cells, fibroblasts, blood and lymphatic vessels, and interstitial extracellular matrix (ECM)] plays a central role in maintaining tissue homeostasis and is a barrier to tumorigenesis (26). Incorrect signals (chemokines, cytokines, reactive oxygen species, lipid mediators, etc.) from an aberrant microenvironment alter tissue homeostasis and initiate/promote tumor growth. Thus, the multiple interactions between stromal and tumor cells are crucial for the initial phases of tumor development.

Prolonged low-grade inflammation or smoldering inflammation is a hallmark of cancer (27, 28). Several cells of the innate and adaptive immune system (macrophages, mast cells, lymphocytes, neutrophils, NK, and NK T cells) are stromal components of the inflammatory microenvironment that can promote the development of experimental and human tumors (29, 30).

## Why are Mast Cells Increased in Tumors?

The presence of mast cells in human tumors, initially reported by Ehrlich (1, 2), was extended by Eugen Westphal (31). Tumor-associated mast cells (TAMCs) are present in the microenvironment of several human solid (32–46) and hematologic tumors (47–55).

Peritumoral and/or intratumoral mast cell density is increased in different types of human cancer (56). Tumor cells produce several chemotactic factors acting on receptors expressed by mast cells. Stem cell factor (SCF) (13, 57), also produced by mast cells (58), activates the mast cell Kit receptor (CD117), vascular endothelial growth factors (VEGFs) act on VEGFR-1 and VEGFR-2 (38, 59), angiopoietin 1 (Ang1) acts on Tie2 receptor (60), and CXCL8/IL-8 acts on CXCR1 and CXCR2 (61). Mast cells express CCR2, CXCR2, and CXCR3, which can be important for TAMC localization because their respective ligands, CCL2, CXCL1, and CXCL10, are produced by human tumors (35, 38). PGE_2_ and histamine are chemotactic for mature mast cells through the engagement of EP_2_ receptor (62, 63) and H_4_R, respectively (64). LTB_4_ may be involved in recruitment of mast cell progenitors from the circulation *via* the activation of BLT1 and BLT2 (65). Finally osteopontin (OP), which is upregulated in human cancer (35), induces mast cell migration (66) and degranulation (35).

## The Contribution of Mast Cells to Cancer is Tumor Dependent

The increasing heterogeneity of different subsets of immune cells (e.g., macrophages, T helper cells, mast cells, neutrophils, NK, NK T cells, etc.), their plasticity, and their reciprocal interactions have complicated the comprehension of the role of the inflammatory microenvironment in tumor initiation and development (29).

A large number of studies have tried to identify the contributory functions of TAMCs in tumor growth. In the majority of studies, TAMCs appear functional—either actively promoting or suppressing tumor development and growth—whereas in a few cases they may be simple inert bystanders. In several studies, mast cells appear to play a pro-tumorigenic role in human (Table 1) and experimental tumors (Table 2). Evidence for an antitumorigenic role for mast cells is provided in Table 3. Studies supporting a non-contributing role of mast cells in tumors are outlined in Table 4.

**Table 1:** **Pro-tumorigenic role of mast cells in human tumors**.

Type of cancer	Mast cell staining	Reference
Angioimmunoblastic T-cell lymphoma	Tryptase	(50)

Bladder	Tryptase	(67)

Colorectal	Giemsa	(68)
	Toluidine blue/tryptase	(69)
	Tryptase	(70–73)

Cutaneous lymphoma	Tryptase	(48)

Esophagus	Toluidine blue	(74)

Follicular lymphoma	Tryptase	(51)

Gastric	Toluidine blue	(75)
	Chymase	(76)
	Tryptase	(77, 78)

Hepatocellular	Tryptase	(79)

Hodgkin’s lymphoma	Tryptase	(53–55)

Lung	Tryptase	(80, 81)
	CD117	(82)

Malignant pleural effusion	May-Gruenwald–Giemsa toluidine blue	(35)

Melanoma	Gene expression/toluidine blue	(83)
	Tryptase	(41, 45)

Merkel cell carcinoma	Tryptase	(33)

Pancreas	Tryptase	(37, 84–86)

Plasmacytoma	Toluidine blue	(47)
	Tryptase	(49)

Prostate	Tryptase	(36, 40, 87, 88)

Splenic marginal zone lymphoma	Tryptase	(52)

Thyroid	Tryptase	(38, 61)

**Table 2:** **Pro-tumorigenic role of mast cells in experimental tumors**.

Type of cancer	Mast cell staining	Reference
Bladder cancer	Toluidine blue	(89)
	Tryptase	(67)

Cholangiocarcinoma	Toluidine blue	(46)

Colon	Toluidine blue/proteases	(90)
	Alcian blue/toluidine blue	(91)
	Chloroacetate esterase	(71)

Cutaneous lymphoma	Toluidine blue	(48)
Hepatocellular	NE	(13)
Malignant pleural effusion	May-Gruenwald–Giemsa/toluidine blue	(35)

Melanoma	NE	(92)
	Alcian blue–safranin	(14)
	NE	(93)
	Gene expression/toluidine blue	(83)

Pancreas	Toluidine blue	(94)
	Tryptase	(84, 85)
	CD117	(95)
	Tryptase	(37)

Plasmacytoma	Toluidine blue	(96)

Prostate	Tryptase/toluidine blue	(40)
	Toluidine blue	(87, 97)

Skin	Chloroacetate esterase/hematoxylin	(98)
	Chloroacetate esterase	(11)
	Chloroacetate esterase/toluidine blue	(99)

Thyroid	Tryptase	(38, 61)

Waldenstrom’s macroglobulinemia	CD117/FcεRI/tryptase	(100)

*NE, not examined*.

**Table 3:** **Antitumorigenic role of mast cells in tumors**.

Type of cancer	Mast cell staining	Reference
**Experimental tumors**		

Intestine	Chloroacetate esterase/chymase	(101)
	Tryptase/CD117	(102)

Melanoma	NE	(103)

Prostate	Toluidine blue	(40)

Skin	Tryptase/CD117	(102)
	Giemsa	(104)

**Human tumors**		

Diffuse large B-cell lymphoma	Tryptase	(105)

Breast cancer	CD117	(106)
	Tryptase	(107)
	Alcian blue/Giemsa	(108)
	CD117	(109)

Colorectal	Tryptase/chymase	(110)

Lung	Tryptase/chymase	(111)

Mesothelioma	Tryptase/chymase	(112)

Melanoma	Tryptase/chymase	(43)

Non-small-cell lung cancer	Tryptase	(113)
	Tryptase/chymase	(114)

Ovarian cancer	Tryptase	(115)

Pancreas	CD117	(71)

Prostate	CD117	(116)

*NE, not examined*.

**Table 4:** **Non-contributing role of mast cells in tumors**.

Type of cancer	Mast cell staining	Reference
**Experimental tumors**		

Colorectal	Wright–Giemsa	(117)

Skin	Toluidine blue/chloroacetate esterase	(118)

**Human tumors**		

Colorectal	Tryptase	(119)

Non-small cell lung cancer	Giemsa	(120)

Renal	Toluidine blue	(121)

In several solid tumors, such as thyroid (38, 61), gastric (75–77, 122), pancreas (37, 84, 85, 94, 95, 123), bladder cancers (67), and Merkel cell carcinoma (33), mast cells always appear to be pro-tumorigenic. Similarly, in several hematologic tumors, such as different types of Hodgkin’s (53–55) and non-Hodgkin’s lymphoma (48, 50, 52), and plasmacytoma (47, 96), mast cells are associated with poor prognosis. There are certain tumors such as breast cancer (106, 107, 109) in which mast cells always appear to play an antitumorigenic role. The role of mast cells in the pathogenesis of human melanomas is still unclear and appears to depend on both the microlocalization of these cells (43) and the subtypes of tumor (83).

**Figure 2:**
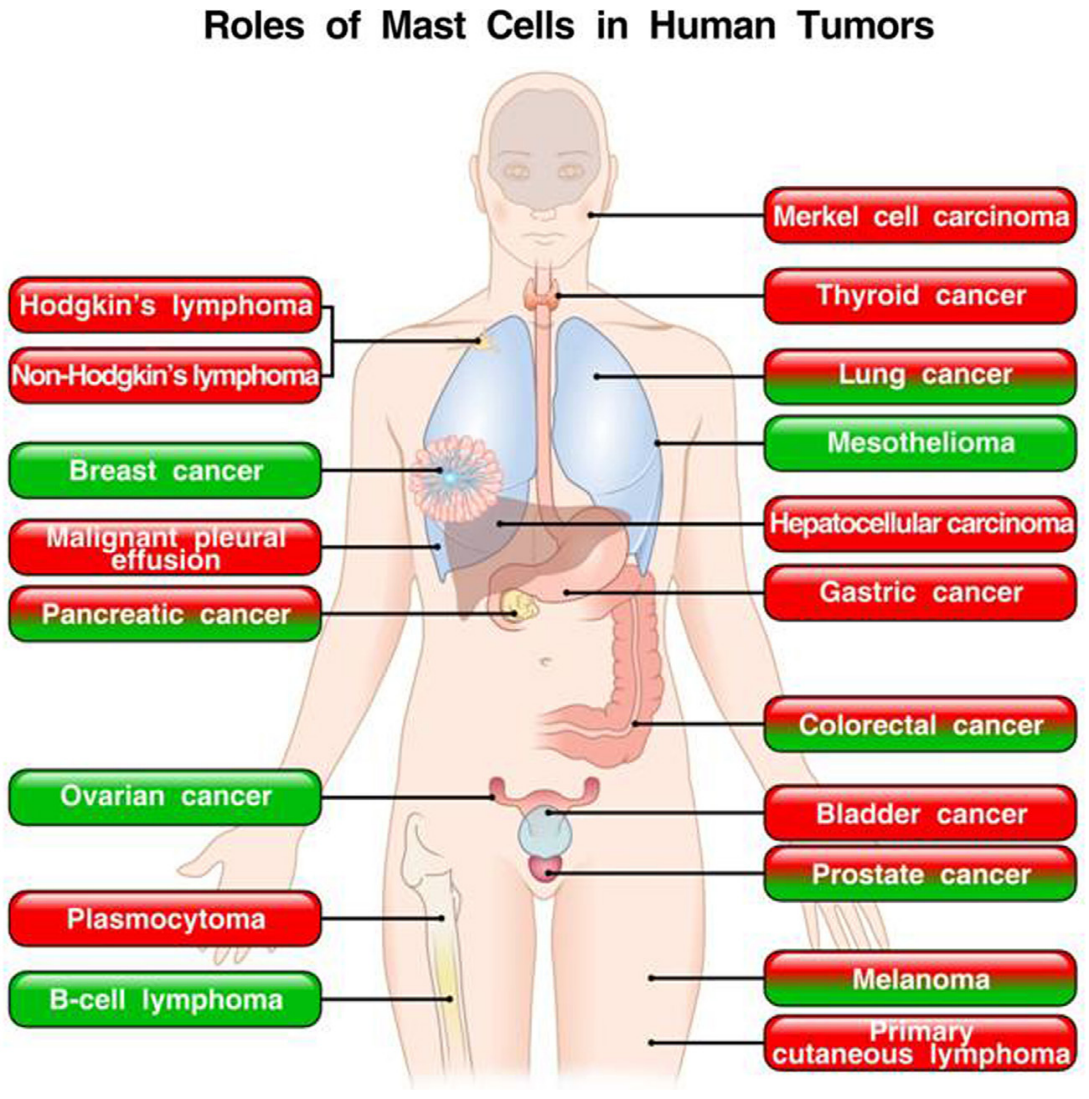
**Roles of mast cells in human tumors**. In red boxes are indicated the tumors in which mast cells play a pro-tumorigenic role. In green boxes are those tumors in which mast cells appear to play a protective role. In mixed red/green boxes are presented tumors in which mast cells play both a pro- and antitumorigenic role in different studies.

These apparently conflicting results are intriguing and suggest that the role of mast cells and their mediators in tumors could be cancer specific. Figure 2 schematically illustrates the role of mast cells in different human tumors.

## Role of TAMCs in Tumor Angiogenesis and Lymphangiogenesis

Angiogenesis, the formation of new blood vessels, is an essential process for supplying growing malignant tissues with essential nutrients and oxygen (124). Lymphangiogenesis, the formation of new lymphatic vessels, is important in the development of metastases (124). Judah Folkman, the father of angiogenesis, suggested that mast cells and macrophages could be attracted by chemotactic molecules produced by tumor cells and could be an important source of proangiogenic factors (125). Several groups have demonstrated that mast cells produce several proangiogenic (VEGF-A, VEGF-B, and FGF-2) (126–130) and lymphangiogenic factors (VEGF-C and -D) (38, 59, 131). In addition, we have found that VEGFs are chemotactic for mast cells (59), indicating that mast cells are a target, in addition to be a source, for VEGFs (132). Several studies have highlighted the association and/or the correlation in human tumors between increased mast cell density and angiogenesis by evaluating the expression of the proangiogenic isoform VEGF-A (42, 45, 70, 80, 96, 123).

The *VEGF-A* gene can be alternatively spliced to form the proangiogenic VEGF-A_165_ and the antiangiogenic VEGF-A_165_b (133). The vast majority of the studies performed so far evaluated only the proangiogenic isoforms, whereas in certain tumors the antiangiogenic VEGF-A_165_b isoform is dominant (134). This finding suggests that the majority of results on VEGF-A plasma levels in cancer need to be reinterpreted or require repeating with tools that can differentiate between the two isoforms of VEGF-A (135). For instance, we have recently demonstrated that human neutrophils, under certain circumstances, can produce both pro- and antiangiogenic isoforms of VEGF-A (136). The role of different pro- and antiangiogenic isoforms of VEGFs produced by TAMCs in primary cancers and in the formation of metastases needs further investigation.

Human mast cells produce different matrix metalloproteinases (e.g., MMP-9) (137) and proteases (tryptase and chymase), which regulate the digestion of ECM favoring the implantation of cancer cells in an aberrant microenvironment (13, 98).

Vascular endothelial growth factor-C, released by melanoma cells (138), TAMs (139), and TAMCs (59), likely represents a major lymphangiogenic factor in this tumor. Mast cells can be found in metastatic lymph nodes of cancer patients (140), and the role of lymphangiogenic factors in the formation of metastasis should be further addressed.

Epithelial-to-mesenchymal transition (EMT) is a mechanism by which tumor cells gain metastatic features and contribute to chemotherapy drug resistance (141, 142). In addition, in the context of tumors, EMT can generate cells with stem-like properties (e.g., stemness) (143). We have demonstrated that mast cells can induce EMT and stem cell features in human cancer through the production of CXCL8/IL-8 (61).

## The Role of Mast Cells Varies According to the Stage of Tumors

A recent study found that low mast cell count in perilesional stroma of deeply invasive melanomas predicted poor survival (43). By contrast, mast cell density was not correlated with prognosis in superficially invasive melanomas. The latter findings suggest that the role of mast cells in melanoma is dependent also on the stage of the tumor. The role(s) of these cells in human and experimental melanoma requires additional studies.

Pittoni et al. found that in prostate cancer mast cells exert different functions according to tumor stage. Mast cells were pro-tumorigenic in the initial stages of prostate cancer by supplying MMP-9 in the microenvironment, but became dispensable at later stages (40, 144).

In stage I non-small-cell lung cancer (NSCLC), but not in stage II, peritumoral but not intratumoral mast cell (tryptase^+^ chymase^+^) density was an independent favorable prognostic factor (111).

Vascular endothelial growth factor-B, an angiogenic factor produced by human macrophages and mast cells (59, 139), could play a role in early colon cancer development at the stage of adenoma formation (145).

## The Role of Mast Cells in Tumors Varies According to Their Microlocalization

The vast majority of initial studies evaluating mast cell density in different cancers did not examine differences between the periphery and the center of tumors. There is increasing evidence that different stages of tumors can be associated with qualitative and quantitative changes in different types of immune cells in the periphery and center of tumors (146, 147). The pro- or antitumorigenic role of mast cells in different types of melanomas remains controversial (83, 148). Siiskonen and collaborators found that tryptase^+^ chymase^+^ mast cells in perilesional stroma of melanoma play a protective role (43). In NSCLC, mast cell infiltration of tumor islets confers a survival advantage independently of tumor stage (113, 114). In another study, it was found that only in stage I NSCLC increased peritumoral mast cells were associated with a better prognosis (111). In pancreatic ductal adenocarcinoma, mast cell density in the intratumoral border zone, but not the peritumoral or the intratumoral center zone, was associated with a worse prognosis (86). In prostate cancer, high intratumoral mast cell density was initially associated with good prognosis (116). Subsequently, it was reported that intratumoral mast cells inhibited tumor growth, whereas peritumoral mast cells stimulated human prostate cancer (36).

Mast cells are increased in patients with both cutaneous T-cell lymphoma and cutaneous B-cell lymphoma compared with normal skin, particularly at the periphery of the tumors. Interestingly, the density of mast cells in the center of tumors was similar to normal skin. The density of peripheral mast cells correlated with disease progression (48).

Collectively, these findings suggest that the microlocalization of mast cells is an important aspect in the initiation and progression of several tumors.

**Figure 3:**
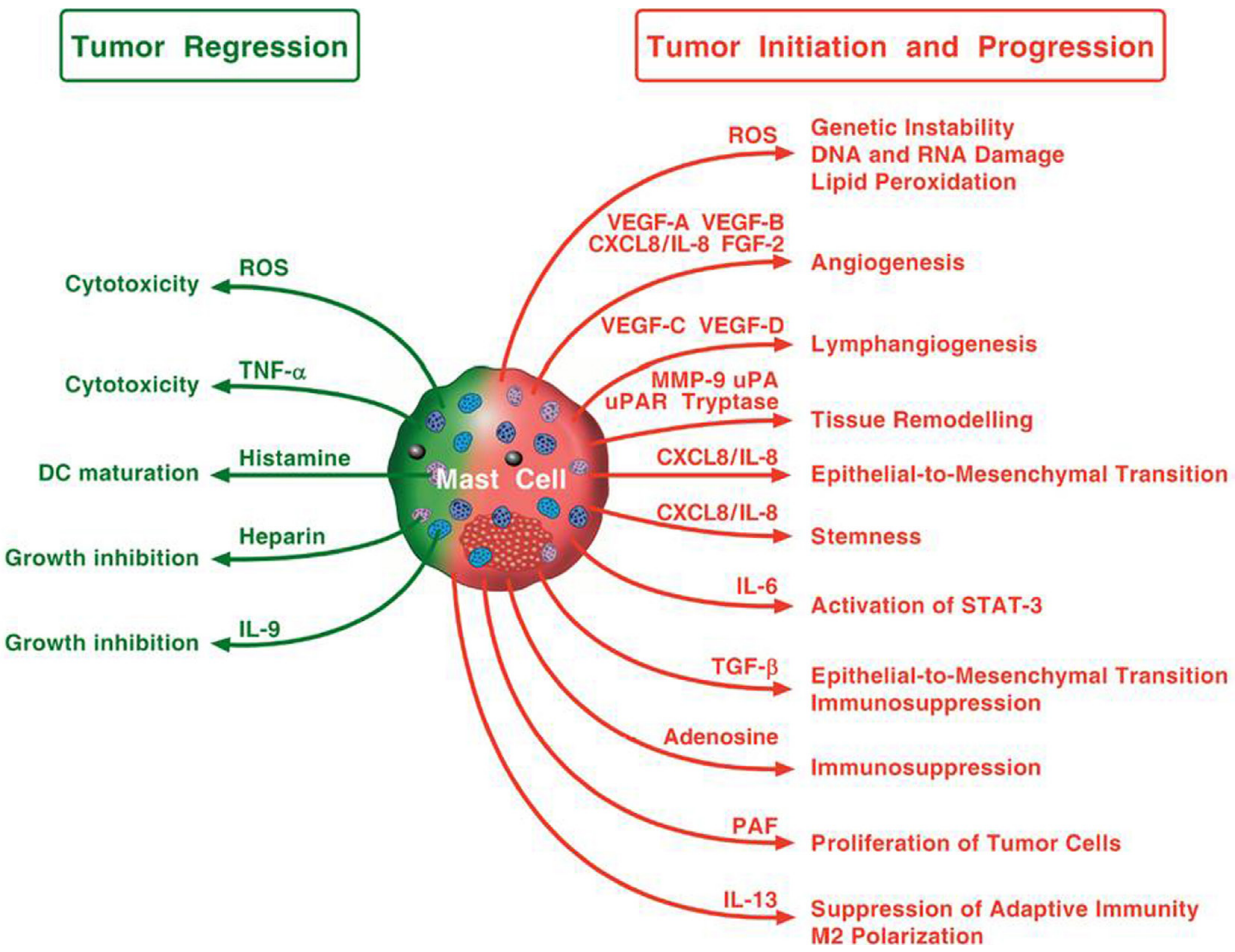
**Possible mechanisms by which mast cells and their mediators may play a pro-tumorigenic or an antitumorigenic role**. Mast cells in tumor microenvironment can promote tumor initiation and progression through the release of ROS, angiogenic and lymphangiogenic factors, and proteases, the induction of epithelial-to-mesenchymal transition and stemness. Mast cells can also activate STAT-3, contribute to immunosuppression and macrophage M2 polarization, and stimulate proliferation of tumor cells. Mast cells can exhibit antitumor activity through direct tumor cell cytotoxicity mediated by ROS and TNF-α or indirectly through the release of heparin, IL-9, and stimulation of dendritic cell maturation.

Figure 3 schematically illustrates the mechanisms by which mast cells and some of their mediators may play a pro-tumorigenic or an antitumorigenic role.

## Which are the Activators of TAMCs in Tumor Microenvironments?

Peritumoral and intratumoral mast cells operate in an inflammatory microenvironment characterized by hypoxia, the accumulation of lactic acid, adenosine, PGE_2_, IFN-γ, and by low pH (149–151). This milieu is likely to influence mast cell recruitment and activation. Mast cells can be recruited by SCF produced by several tumors and by mast cells themselves (13, 58). Mast cells can be recruited by VEGFs and Ang1 produced by tumor and immune cells through the engagement of VEGFR-1/VEGFR-2 and Tie2, respectively, expressed by human mast cells (38, 59, 60).

Hypoxia, a feature of tumor microenvironment (150), activates human mast cells to release IL-6 (152) and VEGF-A (153). Adenosine, produced by tumor cells and mast cells (154), is markedly increased (150) and is an immunosuppressive factor in tumor microenvironment (13). Adenosine potentiates histamine release (155) and the production of angiogenic factors from human mast cells and macrophages (61, 139, 156). Cyclooxygenase 2, overexpressed in tumors (150), generates PGE_2_ which induces angiogenic and lymphangiogenic factors from human mast cells (59). Several chemokines (CXCL1, CXCL10, and CXCL12) can activate mast cells and enhance mast cell secretion of CXCL8/IL-8 (38, 157). Thus, these chemokines can promote angiogenesis/lymphangiogenesis *via* the recruitment of mast cells to the edge of solid tumors.

The impact of IgE-mediated activation of mast cells on tumor development and progression has been investigated (158). Monomeric IgE, in the absence of antigen, induced VEGF-A production from mast cells and increased melanoma growth (8). Increased expression of immunoglobulin free light chains (FLC) was found within stroma of various human cancers. In a murine B16F10 melanoma model, inhibition of FLC-mediated mast cell activation reduced tumor growth (12). Alarmins are upregulated in cancers (159) and can activate mast cells (160). IL-33 is upregulated in squamous cell carcinoma (SCC) (161), and mast cell activation by IL-33 occurs in skin cancers (161). IL-33 induces the production of GM-CSF, CXCL8/IL-8, and VEGF-A from mast cells (128, 162, 163). In addition to the high-affinity receptor for IgE (FcεRI), human mast cells express the IgG receptors FcγRIIA and FcγRI (164, 165). FcγRI is upregulated by IFN-γ which is highly expressed in tumors. In the tumor microenvironment, antitumor IgG immune complexes may activate mast cells (166). OP, upregulated in human cancer (167), is produced by mast cells (66) and induces their migration and degranulation (35, 66). Platelet-activating factor, produced by human mast cells (168), upregulates CXCR4 on mast cells and promotes their migration to lymph nodes (169, 170).

In summary, a plethora of immunologic and non-immunologic factors present in tumor microenvironment can activate TAMCs.

## Mast Cells as a Potential Therapeutic Target in Cancer

Several therapeutic strategies have been envisioned to limit tumor growth by targeting mast cells and their mediators. Mast cells play a pro-tumorigenic role in human bladder cancer through stimulating estrogen receptor β (ERβ) (67). In a murine model of bladder cancer, these authors showed that a selective ERβ antagonist inhibited mast cell-promoted tumor growth. It has been found that mast cells can promote the proliferation of colon cancer *in vivo* (71). Injection of Fcε-PE40 chimeric toxin, which induced mast cell apoptosis, inhibited colon tumor development *in vivo*.

Pharmacologic inhibition of mast cell degranulation by cromolyn inhibited Myc-induced pancreatic islet tumors (94), experimental pancreatic and thyroid cancer (37, 38, 95), and cholangiocarcinoma (46).

Pittoni and collaborators have demonstrated that pharmacologic inhibition by cromolyn and genetic ablation of mast cells inhibited prostate cancer in mice (40). However, mast cells protect from a malignant neuroendocrine tumor. It has been shown that mast cells can promote prostate cancer chemotherapy and radiotherapy resistance *via* modulation of p38/p53/p21. The authors suggested that targeting these signaling pathways may help to suppress chemo- and radiotherapy resistance in prostate cancer (97). In a mouse model, mast cells enhanced prostate cancer growth *via* modulation of androgen receptor and increasing MMP-9 expression (87). The authors suggested that targeting these mast cell-androgen receptor signals may inhibit tumor growth.

The UV wavelengths in sunlight are the prime etiological cause of skin cancers, including basal cell carcinoma and SCC. Exposure to UV affects skin mast cell migration by altering the CXCR4–CXCL12 axis (99). The pharmacological blockage of the CXCR4–CXCL12 pathway inhibited sunlight-induced skin cancer.

Collectively, these findings indicate that mast cells and their mediators deserve focused consideration as therapeutic targets in different types of cancer.

## Outstanding Questions

There is compelling evidence that human mast cells isolated from various anatomical sites respond to different stimuli and release distinct mediators (14, 59, 160, 166, 171). Peritumoral and intratumoral TAMCs are embedded by a wide spectrum of mediators and in close contact with several stromal cells. It will be important to identify the stimuli that can activate TAMCs in different tumor microenvironments. Similarly, it will be important to identify preformed and *de novo* synthesized mediators released *in situ* by TAMCs.

Studies on mast cell biology are routinely conducted at physiological pH and normoxia. By contrast, the metabolic phenotype of tumors is characterized by low pH and areas of either hypoxia or normoxia (150). Tumor-associated macrophages in normoxic tumor tissues express M1 markers, whereas those in hypoxic tumor tissues preferentially express M2 markers (172). These findings caution against the over interpretation of results from studies of whole TAMC populations. It will be of fundamental importance to investigate how hypoxic conditions and metabolism activate/modulate the production of pro-inflammatory and angiogenic/lymphangiogenic factors from TAMCs. Proteomic (173) and lipidomic analyses (174) of mast cells will help to characterize the proangiogenic and antitumorigenic profiles of TAMCs from different human tumors.

Analysis of mast cells in draining lymph nodes and in ectopic lymphoid structures of tumors has only recently begun (35, 43). The role of mast cells in draining lymph nodes, in tertiary lymphoid tissues, and at metastatic sites of different tumors remains to be explored.

IgE has been suggested to play a protective role in tumor growth (21, 158). Additional studies should investigate the role, if any, of IgE-mediated activation of mast cells in different human tumors.

The pro- or antitumorigenic role(s) of mast cells in different human tumors appears to be generally, but not always, cancer specific. We cannot exclude the possibility that subpopulation of TAMCs could play different, even opposite effects in various types/subtypes of tumors.

There is preliminary evidence that peritumoral mast cells (48) play different roles compared to intratumoral mast cells (36, 113, 114). Studies in other experimental and human tumors will clarify whether the microlocalization of mast cells can markedly influence their effects.

Within the last years, gene expression profiling has demonstrated that several individual human cancers (e.g., melanoma, gastric, lung, and breast cancers) are heterogeneous with a spectrum of molecular changes (83, 175–178). The complex heterogeneity (spatial, temporal, intratumor, intertumor) of the tumor microenvironment adds an additional layer of complexity (179, 180). An important task will be to correlate the role of TAMCs in different subtypes of human cancers as defined by genetic markers.

There is recent evidence in melanoma (43), in prostate (40), and in pancreatic cancer (37) that mast cells can play different roles in early and late phases of tumor initiation and growth. This fascinating hypothesis deserves to be further investigated in order to clarify the functional role of TAMCs in the progression of experimental and human cancers.

Two strains of mast cell-deficient mice with mutations affecting Kit, Kit^W/w−v^ (90, 91, 94, 98, 101, 104) and Kit^W−sh/W−sh^ (14, 35, 40, 85, 89), have been extensively used to study the role of mast cells in tumor growth. These mice are profoundly deficient in mast cells and also exhibit several other abnormalities, such as basophil deficiency (181, 182). Recent evidence suggests that basophils can play a role in human pancreatic cancer (183). New Kit-independent mast cell-deficient mice (184) have been used to evaluate the role of mast cells in cutaneous lymphoma (48), malignant pleural effusion (35), and skin cancerogenesis (118). Collectively, results obtained with mast cell-deficient mouse models should be interpreted with caution because even new mouse mutants with unperturbed Kit function also showed some hematological abnormalities (184, 185).

Mast cells are plastic cells: their phenotype depends on their anatomical location and the physiological or pathological context (4, 5, 171). TAMCs are exposed in a hostile tumor environment to increased levels of lactate, PGE_2_, adenosine, IFN-γ, and a low pH (149, 150). This metabolic milieu can profoundly alter mast cell behavior. It has been shown that it is possible to reverse the immunosuppressive and pro-tumoral properties of tumor-associated macrophages (186, 187). A better knowledge of the pro-tumorigenic profile of TAMCs could help to “re-educate” these cells to play an antitumorigenic role.

Tumor cells evade host immune attack by expressing several checkpoint inhibitors, such as programmed cell death-1 (PD-1) ligands (PD-L1 and PD-L2) which inhibit PD-1^+^ lymphocytes in tumor microenvironment (188). Monoclonal antibodies targeting the PD-1/PD-L1 pathway unleash antitumor immunity and have revolutionized the management of a wide spectrum of malignancies (189). Certain cancer cells (e.g., melanoma) express also PD-1, in addition to PD-L1, providing an additional tumor intrinsic mechanism enhancing the pro-tumorigenic effect of PD-1/PD-L1 axis (190). Mouse mast cells highly express PD-L1 and, to a lesser extent, PD-L2 (191). An important task will be to investigate the role of PD-L1^+^ TAMC in tumor microenvironment.

All the above implies that elucidation of the roles of mast cells in different human tumors will demand studies of increasing complexity beyond those assessing merely mast cell density and microlocalization.

## Conclusion

In several human and experimental tumors, mast cells and their mediators play a pro-tumorigenic role. However, in other tumors and even in the same tumor, mast cells seem to play a protective role. These apparently controversial results might reflect differences in stage, grade, and subtypes of tumors, different methods to identify mast cells (e.g., tryptase^+^, chymase^+^, toluidine blue, CD117^+^, Giemsa), or different microanatomical compartment (i.e., peritumoral vs intratumoral) analyzed in the various studies. Whatever the mechanisms, there are many unanswered questions that need to be addressed before we understand whether mast cells are an ally, adversary, or innocent bystander in human cancers.

## Author Contributions

GV, MG, and SL conceived and designed the review. All the authors contributed intellectually and to the writing of the submitted version of the manuscript.

## Conflict of Interest Statement

The authors declare that the research was conducted in the absence of any commercial or financial relationship that could be construed as a potential conflict of interest.
